# Fifteen-year experience of direct bridge with venoarterial extracorporeal membrane oxygenation to heart transplantation

**DOI:** 10.1016/j.xjon.2024.08.014

**Published:** 2024-09-03

**Authors:** Mojgan Laali, Maharajah Ponnaiah, Guillaume Coutance, Guillaume Hekimian, Cosimo D'Alessandro, Pierre Demondion, Guillaume Lebreton, Pascal Leprince

**Affiliations:** a Thoracic and Cardiovascular Surgery Department, Sorbonne Université, APHP, Groupe Hospitalier Pitié-Salpétrière, Institute of Cardiology, Paris, France; b Sorbonne Université, APHP, Groupe Hospitalier Pitié-Salpétrière, ICAN Intelligence and Omics, Institute of Cardiometabolism and Nutrition, Paris, France; c Intensive Care Unit Department, Sorbonne Université, APHP, Groupe Hospitalier Pitié-Salpétrière, Institute of Cardiology, Paris, France

**Keywords:** adult heart transplantation, short-term mechanical circulatory support, extra-corporeal membrane oxygenation

## Abstract

**Objective:**

The study objective was to evaluate outcomes of patients directly bridged with venoarterial extracorporeal membrane oxygenation to heart transplantation.

**Methods:**

A single-center retrospective study was performed on 1152 adult patients undergoing isolated cardiac transplantation between January 2007 and December 2021. Among these, patients bridged with an extracorporeal membrane oxygenation to transplantation (extracorporeal membrane oxygenation group, n = 317) were compared with standard cohorts of patients (no extracorporeal membrane oxygenation group, n = 835). A period analysis (Era 1, 2007-2013, vs Era 2, 2014-2021) was performed.

**Results:**

Median duration of extracorporeal membrane oxygenation support before transplantation in the extracorporeal membrane oxygenation group was 8 days. Recipients of extracorporeal membrane oxygenation group were younger, with a better renal function and a shorter time on the waiting list. They were allocated to younger donors, with a longer ischemic time. The extracorporeal membrane oxygenation group and no extracorporeal membrane oxygenation group showed similar 1-year and 9-year survivals: 79.2% versus 79.4%, *P* = .98, and 56.2% versus 53.9%, *P* = .59, respectively. Period analysis in the extracorporeal membrane oxygenation group showed improved 1- and 9-year survivals in Era 2 compared with Era 1: 82.7% versus 71.1%, *P* = .021 and 60.4% versus 50.5%, *P* = .031, respectively. Era 2 was characterized by a higher rate of patients maintained on extracorporeal membrane oxygenation support after transplantation (92% vs 48%, *P* < .001), inserted mainly by peripheral cannulation (99.51% vs 57%, *P* < .001), for a shorter median duration after transplantation (5 vs 6 days, *P* = .033).

**Conclusions:**

Extracorporeal membrane oxygenation as a direct bridge to heart transplantation shows similar outcomes to standard cohorts of patients. In the extracorporeal membrane oxygenation group, the waiting list time is shorter due to the emergency allocation system, and recipients have no evidence of organ dysfunction at the time of transplantation.

## Graphical abstract

**Figure:**
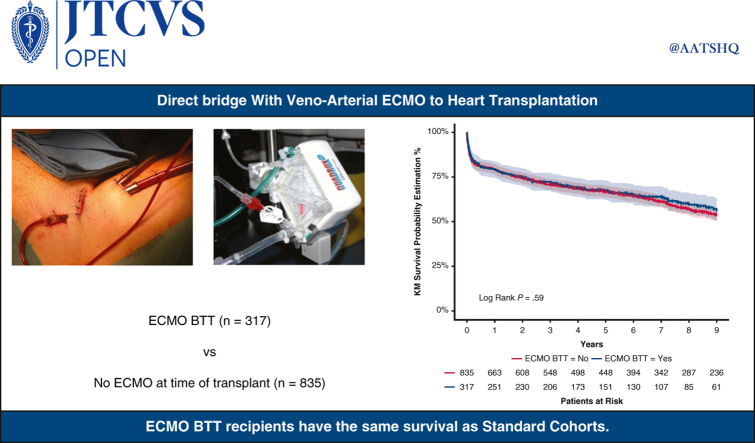

**Figure:**
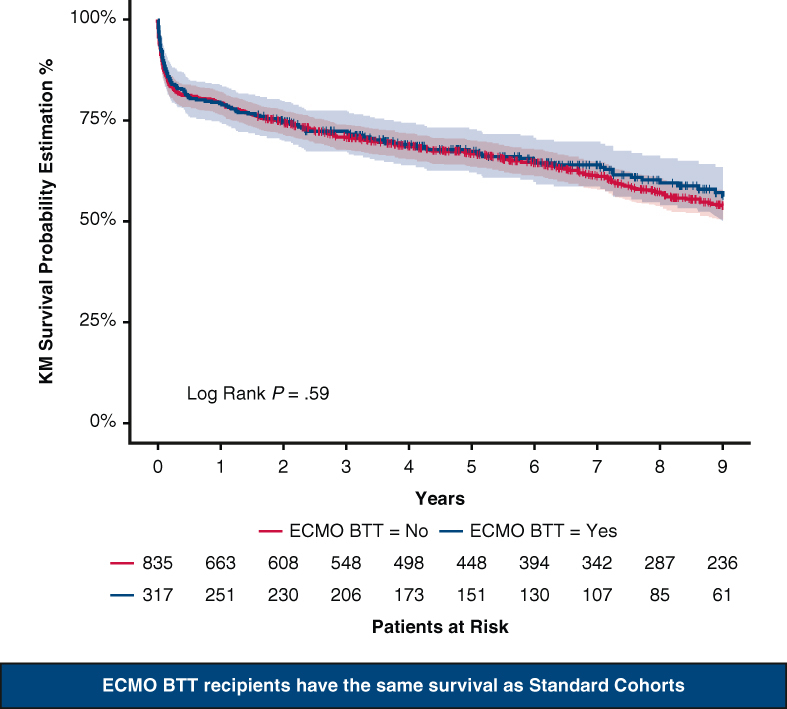
Nine-year Kaplan–Meier survival of patients on ECMO BTT versus standard cohorts.

**Central Message**

ECMO as a direct bridge to HTx shows similar survival to standard cohorts with shorter wait-list time and no evidence of organ dysfunction.

**Perspective**

The controversies exist about post-transplant survival of patients on ECMO directly bridged to HTx. This retrospective study suggests that ECMO as a direct bridge to HTx is a reliable therapeutic option, with similar outcomes as standard cohorts of patients as long as the waiting list is short and the recipient has no evidence of organ dysfunction.

See Discussion on page 304.

Over the last few decades, extracorporeal membrane oxygenation (ECMO) has proven invaluable in the treatment of patients with advanced decompensated heart failure and cardiogenic shock, as a bridge to recovery, or durable mechanical circulatory support (MCS) and heart transplantation (HTx).^1^

On the other hand, in most countries, transplant candidates managed with ECMO are allocated the highest priority status for HTx. As a result, the proportion of recipients receiving ECMO as a direct transition strategy to transplantation has increased in recent years.^2^ Subsequently, the effects of a direct bridge from ECMO to HTx (ECMO bridge to transplant [BTT]) on perioperative complication rates and post-transplant outcomes came under closer scrutiny.^3^

In this group of patients, although some retrospective studies have shown comparable short-term survival after changes in heart allocation policies,^4^^,^^5^ other studies have shown higher rates of renal failure and poorer survival results in the post-transplant period.^6^, ^7^, ^8^ These controversies led us to analyze the experience of our high-volume ECMO implantation center over the past 15 years.

This retrospective study was designed to compare the outcomes and postoperative results of patients on ECMO BTT with the group of patients who are on the waiting list without ECMO support. We also verified the effect of era on ECMO BTT group results.

## Patients and Methods

### Study Population

A single-center retrospective analysis included 1152 consecutive adult undergoing isolated HTx between January 1, 2007, and December 31, 2021. During the study period, 390 patients on ECMO were listed for direct BTT. Among these, 317 patients (81%) underwent transplantation, 57 patients (14.6%) died on the waiting list, 2 patients (0.5%) were removed from the list because of worsening of their pathology, and 6 patients (2%) were removed from the list because of improvement in pathology. Eight recipients (2%) were bridged to ventricular assist devices (VADs) due to absence of allocation. A total of 317 patients on ECMO support directly bridged to transplantation (ECMO group) were compared with the standard cohorts of patients (no ECMO group, n = 835). Only 30% (94 patients) of recipients in the ECMO group were already listed for transplantation and subsequently required ECMO support due to worsening their clinical conditions.

A period analysis (Era 1, 2007-2013 vs Era 2, 2014-2021) was performed to verify the effect of experience on the results. Study follow-up ended on August 1, 2023. Baseline donors, recipients, transplant characteristics, and outcomes and follow-up data were obtained from the prospective national registry “Cristal,” the database of the French regulatory agency for organ procurement, “Agence de BioMédecine.” The CRISTAL database is registered with the national data protection authority (Commission Nationale Informatique et Libertés Registration Number: 363505). An Institutional Review Board grant was released by the ethical committee of the French Society of Thoracic and Cardiovascular Surgery (DELIBERE_CERC-SFCTCV-2024-01-30_32601_Mojgan Laali).

### Definitions and Outcomes

The primary end points were survivals at 1 year and 9 years; the latest available follow-up data were 9 years. Post-transplant ECMO was defined as early if the need for support occurred within the first 48 hours postoperatively; after that, it was defined as secondary. Weaning failure was defined as the need for secondary ECMO or death due to cardiogenic shock after weaning from post-transplant ECMO support.

### Extracorporeal Membrane Oxygenation Characteristics and Technique

The extracorporeal system consisted of polyvinyl chloride tubing, a membrane oxygenator, a centrifugal pump, and either arterial and venous femoral or central right atrial and aortic cannulae. An oxygen/air blender was used to ventilate the membrane oxygenator. When femoral ECMO was instituted, an additional 5F cannula was inserted distally into the superficial femoral artery to prevent possible leg ischemia. Since 2017, femoral ECMO has been inserted percutaneously. If needed, a concomitant IABP was inserted to unload the left ventricle and avoid pulmonary edema. If a central cannulation was chosen, left ventricular venting through the pulmonary artery or left ventricle apex was implemented when there was total absence of cardiac contractility. Patients were anticoagulated with heparin to achieve an activated cephalin time of twice the control.

### Criteria for Post-transplant Extracorporeal Membrane Oxygenation Cannulation

Our criteria for post-transplant ECMO cannulation were the concurrent occurrence of failure to achieve a satisfactory cardiac output (velocity time integral >10 cm) without right ventricular dilatation and central venous pressure above 15 mm Hg despite adequate preload, maximal inotrope support with dobutamine 10 μg/kg/min, norepinephrine 1 μg/kg/min, and inhaled nitric oxide. If these criteria were met at cardiopulmonary bypass (CPB) weaning, even after at least 1 hour of support, immediate transition from CPB to ECMO was made. If the patient was cannulated after chest closure in the operating room or intensive care unit, the cardiac index measurement on the Swan-Ganz catheter and arterial gas values for pH and lactates were also taken into account.

### Criteria for Post-transplant Extracorporeal Membrane Oxygenation Weaning

When a pulsatile arterial waveform had been maintained for at least 24 hours and echocardiographic assessment showed recovery of systolic cardiac function and pulmonary blood oxygenation was not compromised, an ECMO weaning trial was performed by progressively reducing the pump flow to less than 1 L/min (maintaining the minimum speed of 1500 rpm to prevent retrograde flow). Once this was achieved, the arterial and venous lines were clamped for 1 to 2 minutes (after increasing anticoagulation). In this setting, if the left ventricular ejection fraction was more than 35% to 40%, the aortic blood flow time-velocity integral was more than 10 cm, and the cardiac index was more than 2.2 L/min/m^2^, the flow was maintained between 1.5 and 2 L/min for 24 hours. If the patient's hemodynamic status remained stable under these conditions, ECMO was withdrawn.

### Statistical Analyses

Continuous variables were presented as medians (25th-75th percentile), and categorical variables were expressed as frequency (percentages). The Kaplan–Meier method was used to evaluate survival, with the log-rank test for group comparisons. The associations between baseline variables and mortality were analyzed using a Cox proportional hazards regression model, with a preemptive test of the proportional hazard’s assumption. Likewise, the association between baseline variables and primary clinical outcomes was assessed through multivariable logistic regression using a backward conditional method. Variables significant at the 10% level in univariate analysis were included. The overall fit of the models was confirmed by Omnibus tests of model coefficients, and model validation was conducted via receiver operating characteristic curve analysis. Odds ratios were reported with 95% CIs. Extreme Gradient Boosting (XGBoost) was selected for the predictive modeling because of its efficiency and capability in handling complex datasets and its robustness against overfitting.^9^ This ensemble learning method constructs sequential decision trees, with each tree correcting its predecessor's errors, known for its high performance and speed. The dataset was divided into a training set (70%) and a test set (30%) through stratified sampling to preserve the outcome variable's distribution. The XGBoost model was trained on the training set with predefined hyperparameters, including learning rate, tree depth, and number of trees, optimized via cross-validation to mitigate overfitting and enhance generalizability. Model efficacy was assessed on the test set, focusing on accuracy and precision. A figure illustrating the importance of features in predicting the outcome was plotted. Missing data were related to pulmonary vascular resistance in 478 patients. Patients undergoing transplantation in an urgent setting, as well as the majority of patients in the ECMO group, did not undergo right heart catheterization. Therefore, this variable was used only in the descriptive analysis, but not in the analysis of the association between baseline characteristics and mortality. Other missing data (N miss in the Tables 1, E1, and E2) concerned few patients and did not require additional analysis. Statistical analyses were conducted using SPSS version 26.0, and the XGBoost model was implemented with the “xgboost” package in R version 3.5.3.

## Results

### Patients

Recipient and donor characteristics are summarized in Table 1, A and B. The median duration of ECMO support before transplantation in the ECMO group was 8 days [4.00; 13.0]. Recipients in the ECMO group were younger (49.6 [36.4; 58.0] vs 54.1 [44.4; 60.9] years, *P* < .001), with better renal function (estimated glomerular filtration rate [eGFR] 105 [71.2; 151] vs 72 [53.9; 93.9] mL/min, *P* < .001) but higher bilirubin levels (25.0 [15.0; 40.0] vs 14.0 [9.00; 23.0] μmol/L, *P* < .001). Preoperative implantation of an implantable cardioverter defibrillator (ICD) before transplantation was less frequent in recipients in the ECMO group, 116 patients (37%) versus 522 patients (63%) (*P* < .001). Concomitant support with inotropes or intra-aortic balloon pump (IABP) were more frequent in the ECMO group, 264 patients (83%) versus 274 patients (33%) (*P* < .001) and 148 patients (47%) versus 5 patients (0.6%) (*P* < .001), as well as the need for preoperative mechanical ventilation, 41 patients (13%) versus 10 patients (1.2%) (*P* < .001). In the ECMO group, an IABP was placed at the same time as ECMO to prevent left ventricular overload. The 5 patients in the no ECMO group were transferred from a secondary center under IABP, which was maintained until transplantation. The IMPACT score^10^ was higher in the ECMO group (3 [2; 5] vs 12 [10; 14], *P* < .001), surely because of integration of preoperative ECMO support in the score calculation.

**Table 1:** Patient characteristics

A. Preoperative characteristics – Recipient					
Characteristic	N non miss; N miss	Overall population (n = 1152)	No ECMO group (n = 835)	ECMO group (n = 317)	*P*
Age	1152; 0	52.8 [42.5; 60.3]	54.1 [44.4; 60.9]	49.6 [36.4; 58.0]	**<.001**
Weight (kg)	1152; 0	72.0 [63.0; 82.0]	72.0 [63.0; 82.0]	72.0 [63.0; 83.0]	.38
Height (cm)	1152; 0	172 [167; 178]	172 [167; 178]	172 [167; 178]	.82
BMI (kg/m^2^)	1152; 0	24.1 [21.7; 27.0]	24.1 [21.8; 26.9]	24.3 [21.5; 27.4]	.31
BSA (m^2^)	1152; 0	1.86 [1.71; 2.01]	1.85 [1.71; 2.01]	1.86 [1.71; 2.01]	.53
eGFR (mL/min)	1143; 9	77.2 [57.1; 109]	72.3 [53.9; 93.9]	105 [71.2; 151]	**<.001**
Creatinine (μmol/L)	1143; 9	96.0 [74.5; 127]	103 [81.0; 132]	76.0 [54.5; 100]	**<.001**
Bilirubin (μmol/L)	1133; 19	16.0 [10.0; 28.0]	14.0 [9.00; 23.0]	25.0 [15.0; 40.0]	**<.001**
IMPACT score	1123; 29	4.00 [2.00; 9.00]	3.00 [2.00; 5.00]	12.0 [10.0; 14.0]	**<.001**
PVR (Wood units)	674; 478	2.62 [1.80; 3.94]	2.60 [1.80; 3.90]	3.00 [2.00; 4.00]	.21
Waiting time on list (d)	1152; 0	22.3 [5.36; 101]	46.4 [12.4; 144]	4.40 [2.32; 9.33]	**<.001**
Time of preoperative ECMO support (d)	317; 0	8.00 [4.00; 13.0]	-	8.00 [4.00; 13.0]	
Gender	1152; 0				.16
M		880 (76%)	647 (77%)	233 (74%)	
F		272 (24%)	188 (23%)	84 (26%)	
Age >60	1152; 0	306 (27%)	240 (29%)	66 (21%)	**<.001**
Blood type	1152; 0				.97
A		478 (41%)	348 (42%)	130 (41%)	
O		434 (38%)	316 (38%)	118 (37%)	
B		198 (17%)	141 (17%)	57 (18%)	
AB		42 (3.6%)	30 (3.6%)	12 (3.8%)	
Etiology	1152; 0				.21
Idiopathic		551 (48%)	385 (46%)	166 (52%)	
Ischemic		369 (32%)	273 (33%)	96 (30%)	
Congenital		23 (2%)	16 (1.9%)	7 (2.2%)	
Other		209 (18%)	161 (19%)	48 (15%)	
Creatinine clearance class	1143; 9				**<.001**
≥50 mL/min		950 (83%)	664 (80%)	286 (91%)	
30-49 mL/min		162 (14%)	136 (16%)	26 (8.3%)	
<30 mL/min		28 (2.5%)	25 (3%)	3 (0.95%)	
Diabetes	1152; 0	211 (18%)	153 (18%)	58 (18%)	.99
Insulin-dependent	1152; 0	84 (7.3%)	67 (8%)	17 (5.4%)	.12
Prior sternotomy	1152; 0	362 (31%)	273 (33%)	89 (28%)	.13
>1 prior sternotomy	1152; 0	57 (4.9%)	39 (4.7%)	18 (5.7%)	.48
ICD	1152; 0	638 (55%)	522 (63%)	116 (37%)	**<.001**
Vascular disease	1152; 0	86 (7.5%)	76 (9.1%)	10 (3.2%)	**<.001**
Inotrope use	1150; 2	538 (47%)	274 (33%)	264 (83%)	**<.001**
IABP use	1152; 0	153 (13%)	5 (0.6%)	148 (47%)	**<.001**
Impella use	1152; 0	10 (0.87%)	0 (0%)	10 (3.2%)	**<.001**
Model 2.5				7 (2.2%)	
Model 5.0				3 (0.95%)	
Preoperative ECMO use	1152; 0	317 (28%)	-	317 (100%)	
Peripheral	1152; 0	273 (23.7%)	-	273 (86%)	
Central	1152; 0	44 (3.8%)	-	44 (14%)	
MCS use	1152; 0	146 (12.7%)	146 (17%)	-	
CF-VAD		112 (9.7%)	112 (13.4%)	-	
Bi-VAD		2 (0.2%)	2 (0.24%)	-	
TAH		32 (2.8%)	32 (3.8%)	-	
Ventilator use	1152; 0	51 (4.4%)	10 (1.2%)	41 (13%)	**<.001**

B. Preoperative characteristics – Donor					
Characteristic	N non miss; N miss	Overall population (n = 1152)	No ECMO group (n = 835)	ECMO group (n = 317)	*P*
Age	1152; 0	49.0 [38.0; 57.0]	49.0 [39.0; 58.0]	49.0 [37.0; 55.0]	**.04**
Weight (kg)	1152; 0	75.0 [65.0; 85.0]	74.5 [65.0; 85.0]	76.0 [68.0; 85.0]	.18
Height (cm)	1152; 0	172 [165; 180]	172 [165; 180]	174 [168; 180]	**<.01**
BMI (kg/m^2^)	1152; 0	24.8 [22.3; 28.4]	24.9 [22.3; 28.4]	24.8 [22.5; 28.7]	.87
BSA (m^2^)	1152; 0	1.89 [1.75; 2.03]	1.88 [1.75; 2.03]	1.92 [1.80; 2.04]	.052
Weight donor/recipient mismatch	1152; 0	1.00 [–8.00; 13.0]	1.00 [–8.00; 12.2]	3.00 [–8.00; 15.0]	.73
Height donor/recipient mismatch	1152; 0	0 [–6.00; 6.00]	0 [–6.50; 5.00]	0 [–6.00; 8.00]	.074
BMI donor/recipient mismatch	1152; 0	0.759 [–2.82; 4.79]	0.686 [–2.61; 4.65]	0.980 [–3.26; 5.41]	.37
BSA donor/recipient mismatch	1152; 0	0.0209 [–0.174; 0.106]	0.0175 [–0.106; 0.166]	0.0271 [–0.107; 0.214]	.25
Gender	1152; 0				**<.01**
M		743 (64%)	516 (62%)	227 (72%)	
F		409 (36%)	319 (38%)	90 (28%)	
Age, y	1152; 0				.22
<40		304 (26%)	213 (26%)	91 (29%)	
40-49		286 (25%)	206 (25%)	80 (25%)	
50-59		352 (31%)	252 (30%)	100 (32%)	
>60		210 (18%)	164 (20%)	46 (15%)	
Blood type	1152; 0				**<.001**
A		464 (40%)	343 (41%)	121 (38%)	
O		555 (48%)	372 (45%)	183 (58%)	
B		117 (10%)	105 (13%)	12 (3.8%)	
AB		16 (1.4%)	15 (1.8%)	1 (0.32%)	
Cause of death	1152; 0				.96
Vascular		529 (46%)	385 (46%)	144 (45%)	
Trauma		368 (32%)	263 (31%)	105 (33%)	
Anoxia		211 (18%)	155 (19%)	56 (18%)	
Other		44 (3.8%)	32 (3.8%)	12 (3.8%)	
BSA donor/recipient mismatch >0.15	1152; 0	188 (16%)	124 (15%)	64 (20%)	**.029**
Weight donor/recipient mismatch >0.15	1152; 0	350 (30%)	238 (29%)	112 (35%)	**.024**
Male recipient/female donor	1152; 0	248 (22%)	197 (24%)	51 (16%)	**<.01**
PHM ratio	1152; 0	1.01 [0.911; 1.14]	1.01 [0.911; 1.13]	1.01 [0.906; 1.17]	.22
Ischemic time (min)	1152; 0	198 [160; 228]	195 [150; 228]	206 [175; 230]	**<.01**

C. Outcomes					
Post-transplant outcome	N non miss; N miss	Overall population (n = 1152)	No ECMO group (n = 835)	ECMO group (n = 317)	*P*
Early post-transplant ECMO	1152; 0	597 (52%)	347 (42%)	250 (79%)	**<.001**
Peripheral cannulation	597; 0	523 (88%)	294 (85%)	229 (92%)	**.012**
LV unload	597; 0				**<.001**
No unload		447 (75%)	277 (80%)	170 (68%)	
IABP		110 (18%)	40 (12%)	70 (28%)	
Apex LV cannula		32 (5.4%)	22 (6.3%)	10 (4%)	
Double ECMO		4 (0.67%)	4 (1.2%)	0 (0%)	
Impella		4 (0.67%)	4 (1.2%)	0 (0%)	
Implantation at OR	597; 0	542 (91%)	300 (86%)	242 (97%)	**<.001**
Implantation delay (h)	597; 0	0 [0; 0]	0 [0; 1.00]	0 [0; 0]	**<.001**
Support time (d)	518; 5	6.00 [4.00; 9.00]	7.00 [5.00; 10.00]	5.00 [3.00; 7.00]	**<.001**
Weaning rate	597; 0	523 (88%)	291 (84%)	232 (93%)	**<.001**
Weaning failure	523; 0	23 (4.4%)	16 (5.5%)	7 (3%)	.17
Secondary post-transplant ECMO	1151; 1	38 (3.3%)	24 (2.9%)	14 (4.4%)	.19
Surgical revision for bleeding	1152; 0	128 (11%)	84 (10%)	44 (14%)	.065
Pneumonia	1152; 0	287 (25%)	216 (26%)	71 (22%)	.22
Renal replacement therapy	1152; 0	276 (24%)	204 (24%)	72 (23%)	.54
Mediastinitis	1152; 0	140 (12%)	95 (11%)	45 (14%)	.19
CVA	1152; 0	69 (6%)	46 (5.5%)	23 (7.3%)	.26
Length of stay in ICU (d)	1152; 0	15.0 [9.00; 26.0]	14.0 [8.00; 25.0]	17.0 [11.0; 28.0]	.69
30-d mortality	1152; 0	123 (11%)	93 (11%)	30 (9.5%)	.41
1-y mortality	1152; 0	243 (21%)	176 (21%)	67 (21%)	.98

Weight, height, BMI, and BSA mismatches represent the degree of donor oversize relative to the recipient. Bold indicates statistically significant *P* values. *N non miss*, Number non missing; *N miss*, number missing; *ECMO*, extracorporeal membrane oxygenation; *BMI*, body mass index; *BSA*, body surface area; *eGFR*, estimated glomerular filtration rate; *IMPACT*, Index for Mortality Prediction After Cardiac Transplantation; *PVR*, pulmonary vascular resistance; *ICD*, implantable cardioverter defibrillator; *IABP*, intra-aortic balloon pump; *MCS*, mechanical circulatory support; *CF-VAD*, continuous-flow ventricular assist device; *Bi-VAD*, biventricular assist device; *TAH*, total artificial heart; *PHM*, predicted heart mass; *LV*, left ventricle; *OR*, operating room; *CVA*, cerebrovascular accident; *ICU*, intensive care unit.

Based on the allocating policy for patients supported by ECMO, the time on the waiting list was shorter for recipients in the ECMO group (4.4 [2.32; 9.33] vs 46.4 46.4 [12.4; 144] days, *P* < .001). Greater availability for transplantation showed that the donors in the ECMO group were younger (49.0 [37.0; 55.0] vs 49.0 [39.0; 58.0], *P* = .04), less often of female gender, 90 (28%) versus 319 (38%, *P* < .01), and less often with a recipient male/female donor mismatch, 51 (16%) versus 197 (24%, *P* < .01). Although ischemic time was longer in the ECMO group (206 [175; 230] vs 195 [150; 228] minutes *P* < .01), it was still within the limits.

### Outcomes

**Figure 1:**
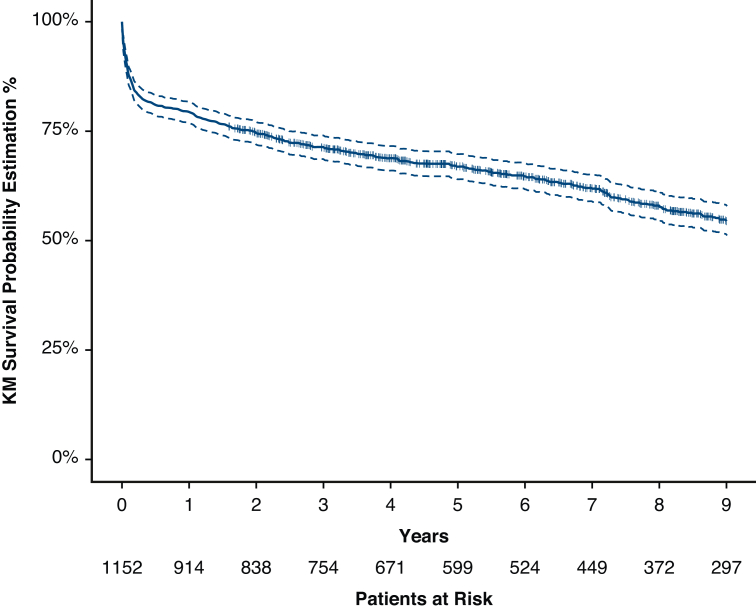
Overall population: Kaplan–Meier survival (95% confidence limits) at 9 years. *KM*, Kaplan–Meier.

Outcomes are summarized in Table 1, C. Overall 1-year mortality was 21%. There were no differences in the causes of death between the 2 groups (Table E1). Factors associated with 9-year mortality are summarized in Table 2. Overall 1-year and 9-year survivals were 79.3% (77.0%; 81.7%) and 54.4% (51.3%; 57.8%), respectively (Figure 1).

**Table 2:** Multivariable analysis for prediction of 9-year mortality

Overall population		
Characteristic	OR (95% CI)	*P*
Recipient age (y, +1)	1.014 [1.003; 1.025]	**.011**
Donor age (y, +1)	1.020 [1.010; 1.030]	**<.001**
Bilirubin μmol/L (+10)	1.007 [1.001; 1.013]	**.027**
Creatinine μmol/L (+10)	1.003 [1.000; 1.005]	.050
Ischemic time (min +1)	1.002 [1.000; 1.005]	.058
Donor BMI (kg/m^2^, +1)	0.952 [0.924; 0.981]	**.001**
BSA donor/recipient mismatch (+0.1)	2.039 [1.040; 3.999]	**.038**
Diabetes	1.562 [1.129; 2.161]	**.007**
Preoperative ECMO use	0.741 [0.539; 1.017]	.064
Preoperative central ECMO use	3.000 [1.514; 5.941]	**.002**
Vascular disease	2.070 [1.271; 3.372]	**.003**

AUC	AUC (95% CI)	*P*
0.713	0.015 [0.684; 0.743]	**<.001**

BSA mismatch represents the degree of donor oversize relative to the recipient. Bold indicates statistically significant *P* values. *BMI*, Body mass index; *BSA*, body surface area; *ECMO*, extracorporeal membrane oxygenation; *AUC*, area under the curve.

**Figure 2:**
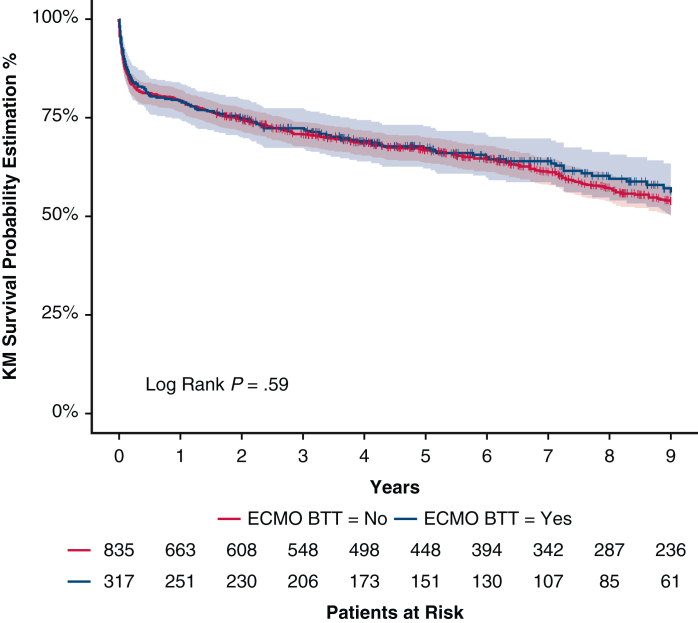
Nine-year Kaplan–Meier survival (95% confidence limits) of ECMO BTT cohort versus standard cohorts. *KM*, Kaplan–Meier; *ECMO*, extracorporeal membrane oxygenation; *BTT*, bridge to transplant.

**Figure 3:**
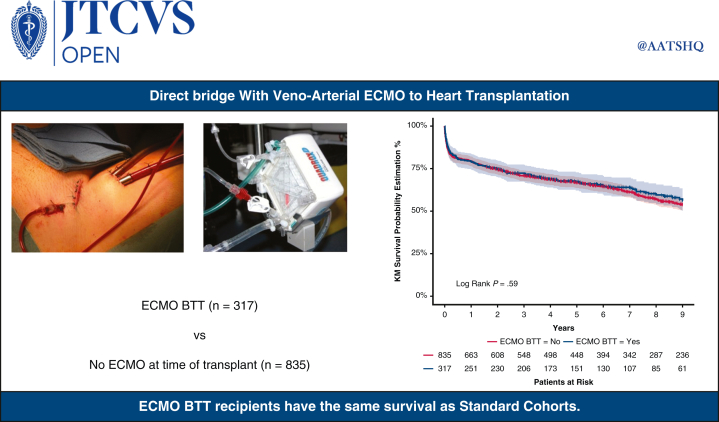
Graphical Abstract. *ECMO*, Extracorporeal membrane oxygenation; *BTT*, bridge to transplant; *KM*, Kaplan–Meier.

One-year and 9-year survivals were similar in both groups: 79.2% (74.8%; 83.8%, ECMO group) versus 79.4% (76.7%; 82.2%, no ECMO group, *P* = .93); 56.2% (49.9%; 63.2%, ECMO group) versus 53.9% (50.2%; 57.8%, no ECMO group, *P* = .59, Figures 2 and 3).

### Period Analysis

Because our policy for ECMO support was changed dramatically in 2013 with more peripheral cannulation and almost systematic use of IABP, a period analysis was performed to verify the effect of experience. Period analyses are shown in Table E2.

In terms of baseline characteristics, period analysis in both groups showed an increase in recipient age, 52.7 [43.7; 60.0, Era 1] versus 55.3 [45.3; 62.0, Era 2] in the no ECMO group (*P* = .022) and 47.6 [31.4; 57.3, Era 1] versus 50.8 [38.2; 58.4, Era 2] in the ECMO group (*P* = .034); the number of recipients with an ICD also increased in the recent era, 228 patients (57%, Era 1) versus 294 patients (67%, Era 2) in the no ECMO group (*P* < .01) and 20 patients (21%, Era 1) versus 96 patients (44%, Era 2) in the ECMO group (*P* < .001); a decrease in preoperative need for mechanical ventilation was also observed, with 10 patients (2.5%, Era 1) versus 0 patients (0%, Era 2) in the no ECMO group (*P* < .001) and 31 patients (32%, Era 1) versus 10 patients (4.5% Era 2) in the ECMO group (*P* < .001). In the no ECMO group, recipients on inotrope support decreased and those on MCS with a continuous-flow VAD increased over time: 145 patients (37%, Era 1) versus 129 patients (30%, Era 2, *P* = .033) and 36 patients (9%, Era 1) versus 76 patients (17%, Era 2, *P* < .001).

From a technical point of view, peripheral cannulation was observed more frequently in the ECMO group in Era 2, 61 patients (63%, Era 1) versus 212 patients (96%, Era 2) (*P* < .001), correlating with a lower rate of prior sternotomy, 51 patients (53%) versus 38 patients (17%) (*P* < .001). Recipients in Era 2 were more often supported with inotropes, 75 patients (77%) versus 189 patients (86%) (*P* = .059), with a more frequent concomitant use of an IABP in 23 patients (24%) versus 125 patients (57%) (*P* < .001). Although not statistically significant, a trend toward increasing donor age was observed in both groups.

In the more recent Era, post-transplant ECMO support was more often inserted earlier in both groups: 108 patients (27%, Era 1) versus 239 patients (55%, Era 2, no ECMO group) (*P* < .001) and 47 patients (48%) versus 203 patients (92% Era 2, ECMO group) (*P* < .001). Because we have changed our technical strategy since 2013, we observed more peripheral cannulation among patients supported with post-transplant ECMO, 62 patients (57%, Era 1) versus 232 patients 97%, Era 2, no ECMO group (*P* < .001) and 27 patients (57%, Era 1) versus 202 patients (99.5%, Era 2, ECMO group, *P* < .001), and more left ventricular unloading by IABP, 1 patient (0.93%, Era 1) versus 39 patients (16%, Era 2, no ECMO group, *P* < .001) and 23 patients (24%, Era 1) versus 125 patients (57%, Era 2, ECMO group, *P* < .001).

**Figure 4:**
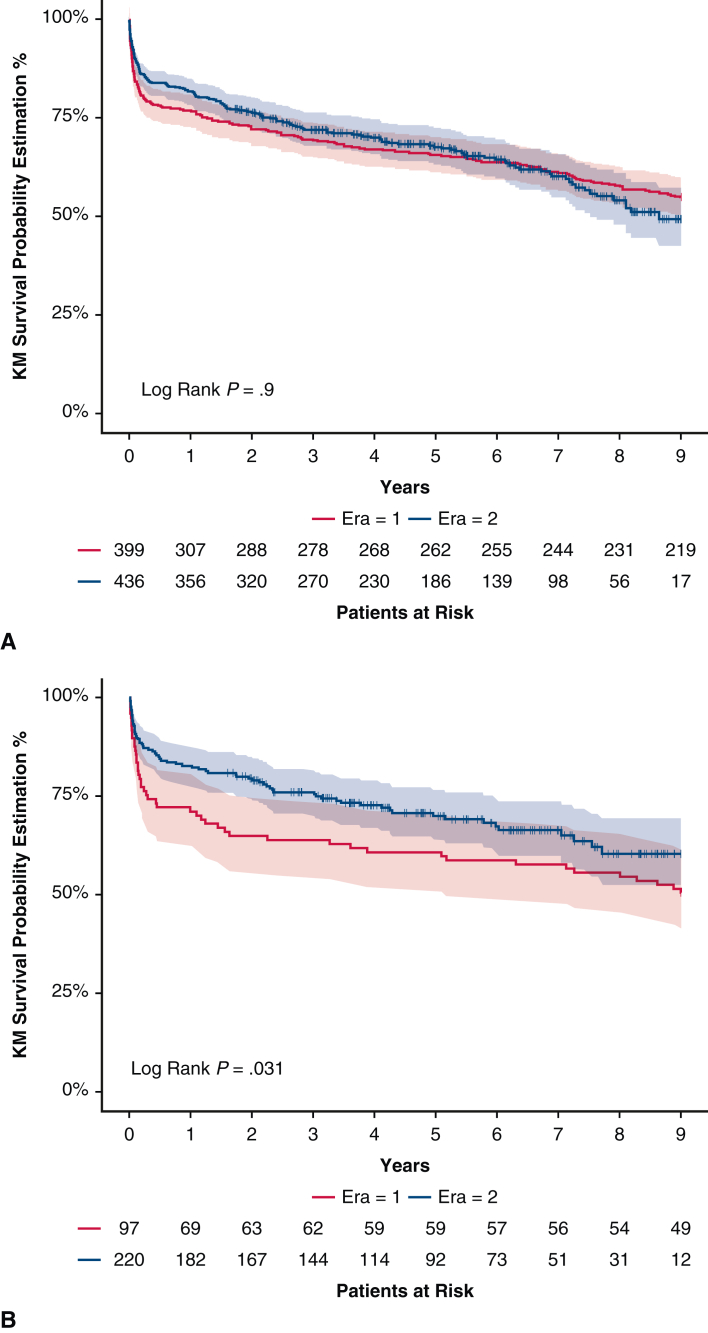
A, Period analysis: Kaplan–Meier survival (95% confidence limits) at 9 years, no ECMO group, Era 1 versus Era 2. B, Period analysis: Kaplan–Meier survival (95% confidence limits) at 9 years, ECMO group, Era 1 versus Era 2. *KM*, Kaplan–Meier.

Median duration of ECMO support after transplantation did not change in the no ECMO group, whereas it decreased in the ECMO group, 6.00 [4.00; 9.00] days in Era 1 versus 5.00 [3.00; 7.00] days, in Era 2, *P* = .033. Early outcomes improved in both groups: The weaning rate was 70%, 76 patients, Era 1 versus 90%, 215 patients, Era 2, in the no ECMO group, *P* < .001, and 81%, 38 patients, Era 1 versus 96%, 194 patients, Era 2, in the ECMO group, *P* < .01. One-year survival improved in both groups: 76.9% (72.9%; 81.2%, Era 1) versus 81.7% (78.1%; 85.4%, Era 2, in the no ECMO group, *P* = .074) and 71.1% (62.7%; 80.7%, Era 1) versus 82.7% (77.9%; 87.9%, Era 2, in the ECMO group, *P* = .021). In period analysis, a reduction in graft failure as a cause of death was noted in both groups through increased use of ECMO after transplantation (Table E1). Nine-year survival improved only in the ECMO group: 54.9% (50.2%; 60.0%, Era 1) versus 49.4% (42.5%; 57.4%, Era 2, in no ECMO group, *P* = .9, Figure 4, *A*) compared with 50.5% (41.5%; 61.5%, Era 1) versus 60.4% (52.5%; 69.5%, Era 2, ECMO group, *P* = .031, Figure 4, *B*).

## Discussion

This retrospective study over 15 years was designed to compare the outcomes of patients bridged directly to HTx by ECMO with standard cohorts of patients. Although according to the International Society for Heart & Lung Transplantation registry, HTx in patients on ECMO represents only 1%,^2^ the rate increases in countries with emergency allocation system.

The percentage of French patients on ECMO at the time of HTx listing has increased from 12% in 2013 to 20% in 2016,^3^ and represents 30% of all HTx in our center, which is a high-volume center. The underrepresentation of ECMO support in International Society for Heart & Lung Transplantation data is also explained by unfavorable outcomes historically reported for these patients.^2^^,^^6^, ^7^, ^8^ The need for ECMO has always been the strongest predictor of 1-year mortality of candidates on the waiting list or delisting due to worsening medical condition.^11^

In France, after a steady improvement in post-transplant outcomes, a significant increase in 1-year mortality was observed for the first time between 2006 and 2008,^3^ which was mainly correlated with HTx performed in ECMO-assisted patients. Therefore, we assumed that ECMO as a direct BTT should be used with moderation and only for those patients with a favorable risk profile.

Our recent results contradict this statement: In our series, patients bridged with ECMO had survival similar to that of the standard cohort of patients. Moreover, machine learning multivariate analysis confirmed that preoperative ECMO does not affect survival (Table 2). Recent reports are consistent with these findings, and they emphasized the importance of a short waiting list for donor allocation. Changes in UNOS policy, by reducing the wait time for patients supported by ECMO before transplantation, resulted in a reduction in the risk of death or clinical deterioration,^12^ with a concomitant improvement in post-transplant mortality.^4^^,^^5^

A recent review described the impact of reducing the length of stay on the waiting list on improving access to organs, as evidenced by an increase in the distance between the donor and transplant centers,^13^ which also resulted in allocation to younger donors with a more favorable gender match.^4^

Because of the allocation policy, we have also seen the same results in our experience, confirming that a short duration on the waiting list is essential to avoid complications due to prolonged ECMO assistance, as noted by Elde and colleagues.^4^ It is important to avoid an unlimited duration of VA-ECMO priority, leading to longer support with increased complications on the waiting list and post-transplant mortality. In the French allocation score system, the score of candidates who are on ECMO decreases from 100% to 0% between 12 and 16 days of ECMO support,^11^ which in itself is longer than the UNOS allocation policy of 7 days.

Because mechanical ventilation and acute renal failure have been identified as major risk factors for poor outcomes after HTx,^14^ recovery of end-organ function is mandatory before HTx candidacy in patients supported by ECMO.^15^ In this regard, the “awake and not intubated” ECMO strategy could decrease ventilation-associated complications, such as muscle deconditioning and ventilator-associated pneumonia.^16^ In our series, only 13% of patients on ECMO were on mechanical ventilation at the time of HTx.

Notably, ECMO support several days before HTx allowed recovery of renal and hepatic dysfunction and increased the number of candidates for immediate bridge to HTx. In the current cohort, eGFR was significantly better in the ECMO group and only 3 patients (0.95%) on ECMO had a creatinine clearance less than 30 mL/min, of whom 2 (0.63%) were already on dialysis at the time of HTx. Concerning liver function, statistical analyses confirm the importance of recipient bilirubin levels as an independent risk factor for mortality.

In cases where ECMO is initiated for acute cardiogenic shock or decompensated chronic heart failure, the patient could be faced with acute pulmonary edema. In our experience since 2004, placing an IABP at the same time could avoid this problem by unloading the left ventricle with lower cost and easier management in the intensive care unit compared with the Impella.^17^ In this series, a small number of patients (10, 3.2%) in the ECMO group were supported concomitantly with the Impella, mostly with the 2.5 L model that was available at the time.

Period analysis of patients in the ECMO group showed a significant improvement in early and midterm survivals over time, consistent with recent reports.^18^ In standard cohorts, we also observe a trend toward increased early survival despite older recipient age and a higher proportion of patients with an ICD, which indicates a longer history of cardiopathy in both groups. In the ECMO group, median recipients eGFR values were slightly higher in Era 2. In addition, the percentage of recipients with eGFR values above 50 mL/min increased significantly over time. On the other hand, the percentage of patients on mechanical ventilation at the time of HTx significantly decreased over the time, and a similar but nonsignificant trend was observed for bilirubin class levels. This period analysis shows how the impact of selecting appropriate candidates for bridge to HTx on ECMO is critical to improving their outcomes.

In the more recent era, post-transplant ECMO support was more often inserted earlier in both groups. Although ECMO support after HTx is relatively high in our service, it could be explained by the following reasons: a significant increase in the age of the recipients and the percentage of recipients aged more than 60 years; the higher number of patients bridged with VADs, which in our experience more often require postoperative ECMO; despite the nonsignificant increase in the median age of the donors, the percentage of donors aged more than 60 years increased significantly; a nearly significant increase in the ischemic time; and finally an improvement in the global management of ECMO, resulting in a more liberal use of this technique.

For patients in the ECMO group, at the beginning of our experience we tried to wean patients off ECMO in the operating room after transplantation, but in most cases it was necessary to reinsert ECMO within 6 hours of the operation.

As shown in Table E2, in Era 1, 15% of patients in the ECMO group, who were weaned at the end of the procedure, required reinsertion of ECMO after leaving the operating room. As we observed in this study,^19^ pretransplant ECMO strongly predicts the need for early post-transplant ECMO support, so in the more recent era we advocate for continuing it after Htx, regardless of allograft function. After recent experience and the positive confirmation of our previous study,^20^ it is our policy to keep patients on ECMO if they arrive in the operating room on ECMO. In such patients in the critical early post-transplant period, continued ECMO support may allow in vivo cardiac allograft reconditioning by reducing right ventricular pressures and preventing right ventricular failure after HTx. Furthermore, emergency ECMO implantation may be particularly challenging in patients with a recent history of ECMO support due to vascular access issues.

This strategy allows ECMO to be removed without reopening the chest, which could reduce the risk of infection. Furthermore, in the case of preoperative peripheral cannulation, as in the majority of cases, we simply left the cannulas in place during HTx, with ECMO resumption after weaning from CPB.

Olivella and colleagues^21^ found a positive effect of peripheral cannulation on mortality in patients requiring post-transplant ECMO support, which was our standard approach in Era 2. This approach is minimally invasive and quickly available even at the bedside if the decision for implantation is taken after the return to intensive care unit and in an emergency context.

In Era 2, left ventricular unloading more often has been achieved by means of an IABP if necessary; this strategy is protective against hydrostatic pulmonary edema by restoring pulsatility and reducing left ventricular afterload.^17^^,^^22^

The results of our study confirm that perioperative management is also central in our direct ECMO to HTx program. We could also hypothesize an immeasurable overall improvement in care over the 2 time periods for both groups. The main interaction between Era effect and ECMO/non-ECMO groups regarding mortality was the higher number of patients with early post-transplant ECMO support, almost systematically in the ECMO group, mainly done by peripheral over central cannulation, with an IABP to unload the left ventricle. This strategy allowed reduced mortality in the non-ECMO group, despite a higher comorbid status.

A final reflection on the bridging strategy should take into account the benefit to moving forward with HTx from ECMO versus ECMO as a bridge to MCS. As shown in a recent EUROMACS report,^23^ we also observed a higher mortality in patients undergoing ECMO as a bridge to MCS. Furthermore, in a strategy with a direct bridge from ECMO to HTx, patients are exposed to the risk of operative mortality only one time. Advocates of ECMO as a bridge to MCS strategy could argue that favorable outcomes have been reported in patients bridged to transplantation with a continuous-flow LVAD versus bridging with ECMO.^6^ On the other hand, the analysis of ELSO registry by Mastoris and colleagues^24^ showed a similar in-hospital mortality between patients bridged with ECMO to HTX or LVAD. In our opinion, both approaches have the flaw of an incomplete analysis of the overall mortality of the strategy adopted: (1) amputating patients on MCS who died before transplant candidacy and (2) patients who died after MCS implantation. However, we could not discuss the superiority of one bridging strategy over another. Patients in the ECMO group did not represent the true intention-to-treat group: Patients who died during the waiting period were not analyzed because they did not receive their transplants. Our work shows the impact of experience in achieving the best results from bridging strategies. If deemed suitable, patients are directly bridged from ECMO to HTx; if the patients are stabilized but unsuitable for a direct HTx or if time on the waitlist exceeds 16 days, they are directed to the bridge-to-transplant or destination therapy with long-term MCS program. In this way, the heart team decision plays an important role in the patient’s registration on the list.

### Study Limitations

As stated in the “Patients and Methods” section, this study carries all the limits that a retrospective design implies. Even if all data were prospectively collected in the database of the French regulatory agency for transplantation with minimal loss of data, our analyses were limited to this variables panel. This is especially true for patients in the ECMO group, who have better renal function and only 13% require mechanical ventilation. We could only hypothesize that their shock state was significantly improved by ECMO support, because we have no data on the severity of shock before ECMO cannulation. For the same reason, patients in the ECMO group were not the true intention-to-treat group: Patients who died during the waiting period were not analyzed because they did not receive their transplants. Although this study did not include data on the severity of shock before ECMO because of the limited data available in the French Transplant Regulation Agency database, the inclusion of patients on the transplant list is discussed in a multidisciplinary committee, providing a homogeneous group of patients, but it is clear that the missing data concerning patients who died under ECMO are a limitation of this study.

## Conclusions

This study represents the largest single-center series confirming that direct bridge to HTx is a reliable therapeutic option for patients on temporary circulatory support with isolated heart failure on ECMO. They present similar outcomes as standard cohorts, provided the waiting list is short and the recipient has no evidence of organ dysfunction. In our opinion, temporary peripheral ECMO support should be pursued after transplantation.

### Webcast

You can watch a Webcast of this AATS meeting presentation by going to: https://www.aats.org/resources/fifteen-years-experience-of-di-7129.

**Figure:**
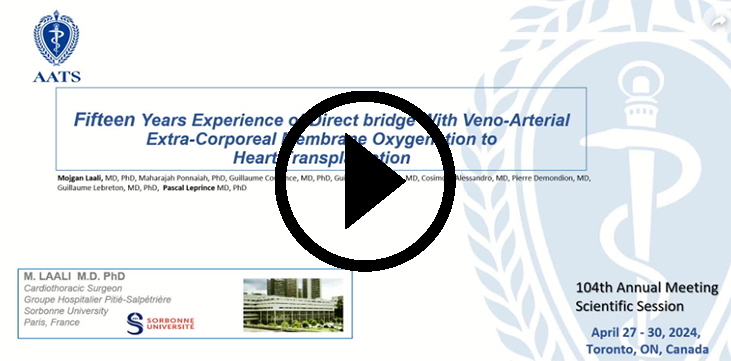

## Conflict of Interest Statement

The authors reported no conflicts of interest.

The *Journal* policy requires editors and reviewers to disclose conflicts of interest and to decline handling or reviewing manuscripts for which they may have a conflict of interest. The editors and reviewers of this article have no conflicts of interest.

## Figures and Tables

**Table E1:** Causes of death at 1 year

Cause of death	N non miss; N miss	Overall population (n = 243)	Group no ECMO (n = 176)	Group ECMO (n = 67)	*P*
Cause of death	243; 0				
Bleeding		10 (4.1%)	5 (2.8%)	5 (7.5%)	.14
Cancer		4 (1.6%)	3 (1.7%)	1 (1.5%)	1
MOF		16 (6.6%)	14 (8%)	2 (3%)	.25
CVA		18 (7.4%)	14 (8%)	4 (6%)	.79
Graft failure		31 (13%)	22 (12%)	9 (13%)	.85
Sepsis		49 (20%)	36 (20%)	13 (19%)	.86
Other		115 (47%)	82 (47%)	33 (49%)	.71

Period analysis – Overall population				
Cause of death	N non miss; N miss	Era 1 (n = 121)	Era 2 (n = 122)	*P*
Cause of death	243; 0			
Bleeding		6 (5%)	4 (3.3%)	.54
Cancer		1 (0.83%)	3 (2.5%)	.62
MOF		14 (12%)	2 (1.6%)	**<.01**
CVA		9 (7.4%)	9 (7.4%)	.99
Graft failure		24 (20%)	7 (5.7%)	**<.001**
Sepsis		30 (25%)	19 (16%)	.073
Other		37 (31%)	78 (64%)	**<.001**

Period analysis – Group no ECMO				
Cause of death	N non miss; N miss	Group no ECMO Era 1 (n = 93)	Group no ECMO Era 2 (n = 83)	*P*
Cause of death	176; 0			
Bleeding		3 (3.2%)	2 (2.4%)	1
Cancer		1 (1.1%)	2 (2.4%)	.6
MOF		13 (14%)	1 (1.2%)	**<.01**
CVA		8 (8.6%)	6 (7.2%)	.74
Graft failure		19 (20%)	3 (3.6%)	**<.001**
Sepsis		23 (25%)	13 (16%)	.14
Other		26 (28%)	56 (67%)	**<.001**

Period analysis – Group ECMO				
Cause of death	N non miss; N miss	Group ECMO Era 1 (n = 28)	Group ECMO Era 2 (n = 39)	*P*
Cause of death	176; 0			
Bleeding		3 (11%)	2 (5.1%)	.64
Cancer		0 (0%)	1 (2.6%)	1
MOF		1 (3.6%)	1 (2.6%)	1
CVA		1 (3.6%)	3 (7.7%)	.63
Graft failure		5 (18%)	4 (10%)	.47
Sepsis		7 (25%)	6 (15%)	.33
Other		11 (39%)	22 (56%)	.17

Bold indicates statistically significant *P* values. *N non miss*, Number non missing; *N miss*, number missing; *ECMO*, extracorporeal membrane oxygenation; *MOF*, multiorgan failure; *CVA*, cerebrovascular accident.

**Table E2:** Period analysis

Preoperative characteristics – Group no ECMO, recipient				
Characteristic	N non miss; N miss	Group no ECMO Era 1 (n = 399)	Group no ECMO Era 2 (n = 436)	*P*
Age	835; 0	52.7 [43.7; 60.0]	55.3 [45.3; 62.0]	**.022**
Weight (kg)	835; 0	71.0 [62.0; 80.0]	72.5 [64.0; 83.0]	**.011**
Height (cm)	835; 0	171 [166; 177]	173 [168; 178]	**.029**
BMI (kg/m^2^)	835; 0	24.1 [21.6; 26.6]	24.2 [21.9; 27.4]	.4
BSA (m^2^)	835; 0	1.84 [1.70; 1.98]	1.87 [1.74; 2.02]	**.032**
eGFR (mL/min)	828; 7	73.0 [55.4; 95.0]	71.4 [53.5; 92.0]	.46
Creatinine (μmol/L)	828; 7	101 [80.0; 130]	105 [83.0; 132]	.83
Bilirubin (μmol/L)	817; 18	0.819 [0.526; 1.40]	0.760 [0.468; 1.29]	**.021**
IMPACT score	808; 27	3.00 [2.00; 5.00]	3.00 [2.00; 5.00]	.64
PVR (Wood units)	577; 258	2.70 [1.91; 4.00]	2.60 [1.80; 3.80]	.56
Waiting time on list (d)	835; 0	27.9 [6.59; 112]	18.3 [4.59; 89.7]	.64
Gender	835; 0			.42
M		314 (79%)	333 (76%)	
F		85 (21%)	103 (24%)	
Age >60 y	835; 0	99 (25%)	141 (32%)	**.016**
Blood type	835; 0			.16
A		165 (41%)	183 (42%)	
O		156 (39%)	160 (37%)	
B		59 (15%)	82 (19%)	
AB		19 (4.8%)	11 (2.5%)	
Etiology	835; 0			.19
Idiopathic		177 (44%)	208 (48%)	
Ischemic		143 (36%)	130 (30%)	
Congenital		5 (1.3%)	11 (2.5%)	
Other		74 (19%)	87 (20%)	
Diabetes	835; 0	74 (19%)	79 (18%)	.87
Insulin-dependent	835; 0	40 (10%)	27 (6.2%)	**.042**
Creatinine clearance class	828; 7			.92
≥50 mL/min		319 (81%)	345 (80%)	
30-49 mL/min		65 (16%)	71 (17%)	
<30 mL/min		11 (2.8%)	14 (3.3%)	
Pretransplant dialysis	835; 0	2 (0.81%)	4 (0.92%)	1
Bilirubin class	817; 18			.14
0-0.99 mg/L		211 (55%)	270 (62%)	
1-1.99 mg/L		120 (31%)	128 (29%)	
2-3.99 mg/L		44 (12%)	34 (7.8%)	
≥4 mg/L		6 (1.6%)	4 (0.92%)	
Prior sternotomy	835; 0	124 (31%)	149 (34%)	.34
>1 prior sternotomy	835; 0	18 (4.5%)	21 (4.8%)	.83
ICD	835; 0	228 (57%)	294 (67%)	**<.01**
Vascular disease	835; 0	34 (8.5%)	42 (9.6%)	.58
Inotrope use	835; 0	145 (37%)	129 (30%)	**.033**
MCS use	835; 0	52 (13%)	94 (22%)	**<.01**
CF-VAD		36 (9%)	76 (17%)	**<.001**
Bi-VAD		2 (0.05%)	0 (0%)	
TAH		14 (3.5%)	18 (4.1%)	.64
Ventilator use	835; 0	10 (2.5%)	0 (0%)	**<.001**

Preoperative characteristics – Group no ECMO, donor				
Characteristic	N non miss; N miss	Group no ECMO Era 1 (n = 399)	Group no ECMO Era 2 (n = 436)	*P*
Age	835; 0	48.0 [39.0; 56.0]	51.0 [40.0; 59.0]	.14
Weight (kg)	835; 0	75.0 [65.0; 85.0]	75.0 [65.0; 85.0]	.48
Height (cm)	835; 0	172 [166; 180]	172 [165; 178]	.55
BMI (kg/m^2^)	835; 0	24.7 [22.4; 27.8]	25.0 [22.2; 28.7]	.41
BSA (m^2^)	835; 0	1.88 [1.75; 2.03]	1.88 [1.74; 2.04]	.7
Weight donor/recipient mismatch	835; 0	2.00 [–5.00; 13.0]	0 [–9.00; 12.0]	.11
Height donor/recipient mismatch	835; 0	1.00 [–5.00; 7.00]	−1.00 [–8.00; 4.00]	**<.01**
BMI donor/recipient mismatch	835; 0	0.802 [–2.26; 4.65]	0.418 [–2.85; 4.64]	.95
BSA donor/recipient mismatch	835; 0	0.0340 [–0.0774; 0.177]	−0.0135 [–0.134; 0.148]	**.011**
Gender	835; 0			
M		255 (64%)	261 (60%)	.23
F		144 (36%)	175 (40%)	
Age, y	835; 0			**.0492**
<40		105 (26%)	108 (25%)	
40-49		108 (27%)	98 (22%)	
50-59		123 (31%)	129 (30%)	
>60		63 (16%)	101 (23%)	
Blood type	835; 0			.23
A		159 (40%)	184 (42%)	
O		180 (45%)	192 (44%)	
B		49 (12%)	56 (13%)	
AB		11 (2.8%)	4 (0.92%)	
Cause of death	835; 0			**.011**
Vascular		201 (50%)	184 (42%)	
Trauma		128 (32%)	135 (31%)	
Anoxia		58 (15%)	97 (22%)	
Other		12 (3%)	20 (4.6%)	
BSA donor/recipient mismatch >0.15	835; 0	62 (16%)	62 (14%)	.59
Weight donor/recipient mismatch >0.15	835; 0	122 (31%)	116 (27%)	.2
Male recipient/female donor	835; 0	98 (25%)	99 (23%)	.53
PHM ratio	835; 0	1.04 [0.912; 1.15]	0.992 [0.911; 1.11]	.07
Ischemic time (min)	835; 0	193 [134; 230]	198 [166; 224]	.062

Outcomes – Group no ECMO				
Post-transplant outcome	N non miss; N miss	Group no ECMO Era 1 (n = 399)	Group no ECMO Era 2 (n = 436)	*P*
Secondary post-transplant ECMO	834; 1	18 (4.5%)	6 (1.4%)	**<.01**
Early post-transplant ECMO	835; 0	108 (27%)	239 (55%)	**<.001**
Peripheral cannulation	347; 0	62 (57%)	232 (97%)	**<.001**
LV unload	347; 0			**<.001**
No unload		85 (79%)	192 (80%)	
IABP		1 (0.93%)	39 (16%)	
Apex LV cannula		21 (19%)	1 (0.42%)	
Double ECMO		1 (0.93%)	3 (1.3%)	
Impella		0 (0%)	4 (1.7%)	
Implantation at OR	347; 0	83 (77%)	217 (91%)	**<.001**
Implantation delay (h)	347; 0	0 [0; 2.00]	0 [0; 0]	**<.01**
Support time (d)	288; 3	7.00 [4.00; 10.0]	7.00 [5.00; 10.0]	.67
Weaning rate	347; 0	76 (70%)	215 (90%)	**<.001**
Weaning failure	291; 0	6 (7.9%)	10 (4.7%)	.38
1-y mortality	835; 0	93 (23%)	83 (19%)	.13

Preoperative characteristics – Group ECMO, recipient				
Characteristic	N non miss; N miss	Group ECMO Era 1 (n = 97)	Group ECMO Era 2 (n = 220)	*P*
Age	317; 0	47.6 [31.4; 57.3]	50.8 [38.2; 58.4]	**.034**
Weight (kg)	317; 0	71.0 [60.0; 80.0]	73.0 [64.0; 84.0]	.14
Height (cm)	317; 0	172 [165; 178]	173 [168; 178]	.3
BMI (kg/m^2^)	317; 0	23.9 [21.0; 26.4]	24.4 [21.6; 27.7]	.57
BSA (m^2^)	317; 0	1.83 [1.68; 1.97]	1.89 [1.73; 2.01]	**.038**
eGFR (mL/min)	315; 2	101 [64.5; 156]	106 [74.3; 147]	.58
Creatinine (μmol/L)	315; 2	78.0 [52.8; 112]	74.0 [55.0; 96.0]	.15
Bilirubin (μmol/L)	316; 1	1.40 [0.877; 2.18]	1.46 [0.877; 2.40]	.8
IMPACT score	315; 2	12.0 [10.0; 14.0]	13.0 [10.0; 14.0]	.51
PVR (Wood units)	97; 220	3.50 [2.30; 4.40]	2.65 [1.87; 4.00]	.26
Waiting time on ECMO support (d)	317; 0	7.00 [3.00; 18.0]	8.00 [5.00; 12.0]	.082
Waiting time on list (d)	317; 0	4.49 [2.42; 9.35]	4.38 [2.31; 9.30]	.19
Gender	317; 0			.46
M		74 (76%)	159 (72%)	
F		23 (24%)	61 (28%)	
Age >60 y	317; 0	16 (16%)	50 (23%)	.21
Blood type	317; 0			.38
A		42 (43%)	88 (40%)	
O		39 (40%)	79 (36%)	
B		12 (12%)	45 (20%)	
AB		4 (4.1%)	8 (3.6%)	
Etiology	317; 0			.44
Idiopathic		45 (46%)	121 (55%)	
Ischemic		15 (15%)	33 (15%)	
Congenital		34 (35%)	62 (28%)	
Other		3 (3.1%)	4 (1.8%)	
Diabetes	317; 0	15 (15%)	43 (20%)	.39
Insulin-dependent	317; 0	4 (4.1%)	13 (5.9%)	.52
Creatinine clearance class	315; 2			**.022**
≥50 mL/min		81 (84%)	205 (94%)	
30-49 mL/min		13 (14%)	13 (5.9%)	
<30 mL/min		2 (2.1%)	1 (0.46%)	
Pretransplant dialysis	317; 0	1 (1.9%)	1 (0.45%)	.35
Bilirubin class	316; 1			.071
0-0.99 mg/L		29 (30%)	70 (32%)	
1-1.99 mg/L		41 (43%)	73 (33%)	
2-3.99 mg/L		16 (17%)	63 (29%)	
≥4 mg/L		10 (10%)	14 (6.4%)	
Prior sternotomy	317; 0	51 (53%)	38 (17%)	**<.001**
>1 prior sternotomy	317; 0	5 (5.2%)	13 (5.9%)	.79
ICD	317; 0	20 (21%)	96 (44%)	**<.001**
Vascular disease	317; 0	6 (6.2%)	4 (1.8%)	.073
Inotrope use	317; 0	75 (77%)	189 (86%)	.059
IABP use	317; 0	23 (24%)	125 (57%)	**<.001**
Preoperative ECMO use	317; 0			**<.001**
Peripheral		61 (63%)	212 (96%)	
Central		36 (37%)	8 (3.6%)	
Ventilator use	317; 0	31 (32%)	10 (4.5%)	**<.001**

Preoperative characteristics – Group ECMO, donor				
Characteristic	N non miss; N miss	Group ECMO Era 1 (n = 97)	Group ECMO Era 2 (n = 220)	*P*
Age	317; 0	46.0 [36.0; 55.0]	49.0 [37.0; 55.2]	.13
Weight (kg)	317; 0	75.0 [65.0; 85.0]	76.0 [68.4; 85.6]	.29
Height (cm)	317; 0	172 [167; 179]	175 [169; 180]	.47
BMI (kg/m^2^)	317; 0	25.0 [22.9; 27.8]	24.8 [22.4; 29.3]	.48
BSA (m^2^)	317; 0	1.92 [1.78; 2.03]	1.93 [1.80; 2.05]	.3
Weight donor/recipient mismatch	317; 0	−5.00 [–17.0; 6.00]	−2.00 [–15.0; 9.62]	.58
Height donor/recipient mismatch	317; 0	0 [–8.00; 5.00]	0 [–7.25; 6.00]	.63
BMI donor/recipient mismatch	317; 0	−1.24 [–5.98; 2.24]	−0.923 [–4.72; 3.45]	.51
BSA donor/recipient mismatch	317; 0	−0.0603 [–0.252; 0.0809]	−0.0234 [–0.202; 0.128]	.27
Gender	317; 0			
M				
F				
Age, y	317; 0			.23
<40		30 (31%)	61 (28%)	
40-49		30 (31%)	50 (23%)	
50-59		27 (28%)	73 (33%)	
>60		10 (10%)	36 (16%)	
Blood type	317; 0			.29
A		32 (33%)	89 (40%)	
O		63 (65%)	120 (55%)	
B		2 (2.1%)	10 (4.5%)	
AB		0 (0%)	1 (0.45%)	
Cause of death	317; 0			.11
Vascular		47 (48%)	97 (44%)	
Trauma		35 (36%)	70 (32%)	
Anoxia		10 (10%)	46 (21%)	
Other		5 (5.2%)	7 (3.2%)	
BSA donor/recipient mismatch >0.15	317; 0	24 (25%)	40 (18%)	.18
Weight donor/recipient mismatch >0.15	317; 0	40 (41%)	72 (33%)	.14
Male recipient/female donor	317; 0	18 (19%)	33 (15%)	.43
PHM ratio	317; 0	1.04 [0.896; 1.19]	1.01 [0.917; 1.17]	.91
Ischemic time (min)	317; 0	211 [180; 230]	200 [174; 229]	.21

Outcomes – Group ECMO				
Post-transplant outcome	N non miss; N miss	Group ECMO Era 1 (n = 97)	Group ECMO Era 2 (n = 220)	*P*
Secondary post-transplant ECMO	317; 0	5 (5.2%)	9 (4.1%)	.77
Early post-transplant ECMO	317; 0	47 (48%)	203 (92%)	**<.001**
Peripheral cannulation	250; 0	27 (57%)	202 (99.5%)	**<.001**
LV unload	250; 0			**<.001**
No unload		37 (79%)	133 (66%)	
IABP		1 (2.1%)	69 (34%)	
Apex LV cannula		9 (19%)	1 (0.49%)	
Double ECMO		--	--	
Impella		--	--	
Implantation at OR	250; 0	40 (85%)	202 (100%)	**<.001**
Implantation delay (h)	250; 0	0 [0; 0.500]	0 [0; 0]	**.015**
Support time (d)	232; 0	6.00 [4.00; 9.00]	5.00 [3.00; 7.00]	**.033**
Weaning rate	232; 0	38 (81%)	194 (96%)	**<.01**
Weaning failure	232; 0	1 (2.6%)	6 (3.1%)	1
1-y mortality	317; 0	28 (29%)	39 (18%)	**.025**

Weight, height, BMI, and BSA mismatches represent the degree of donor oversize relative to the recipient. Bold indicates statistically significant *P* values. *N non miss*, Number non missing; *N miss*, number missing; *ECMO*, extracorporeal membrane oxygenation; *BMI*, body mass index; *BSA*, body surface area; *eGFR*, estimated glomerular filtration rate; *IMPACT*, Index for Mortality Prediction After Cardiac Transplantation; *PVR*, pulmonary vascular resistance; *ICD*, implantable cardioverter defibrillator; *MCS*, mechanical circulatory support; *CF-VAD*, continuous-flow ventricular assist device; *Bi-VAD*, biventricular assist device; *TAH*, total artificial heart; *PHM*, predicted heart mass; *LV*, left ventricle; *IABP*, intra-aortic balloon pump; *OR*, operating room.
